# Thyroid Storm With Myocardial Injury Mimicking Acute Coronary Syndrome: A Case Report and Literature Review

**DOI:** 10.7759/cureus.99338

**Published:** 2025-12-15

**Authors:** Aoumar G Chamma, Wendy Saliba, Linda Chamma

**Affiliations:** 1 Cardiology, University of Balamand, Beirut, LBN; 2 Urology, Lebanese University, Beirut, LBN

**Keywords:** acute heart failure, endocrine emergency, graves´disease, hyperthyroidism, iodinated contrast–induced thyrotoxicosis, thyroid-storm

## Abstract

Thyroid storm presents a fatal medical emergency that develops when thyrotoxicosis advances to its most critical stage. A 50-year-old male patient with no known thyroid problems presented to the hospital complaining of several episodes of diarrhea, tachycardia, and chest pain with ECG changes suggestive of ischemia. Laboratory tests confirmed thyrotoxicosis with a low level of thyroid-stimulating hormone, while free T4 and T3 levels were significantly elevated. Further workup revealed Graves’ disease (positive anti-thyroid peroxidase antibody results and elevated radioactive iodine uptake). The patient received intensive treatment in the ICU, including beta-blockade therapy, anti-thyroid medications, iodine solution, corticosteroids, and supportive medical care. Despite initial concerns for acute coronary syndrome due to troponin elevation and ST depressions, coronary angiography showed no obstructive disease. This indicates a type II myocardial infarction from supply-demand mismatch. The patient underwent radioactive iodine ablation as a final treatment after achieving stability. This case highlights that thyroid storm encompasses symptoms that mimic acute cardiac crisis, thus underscoring the importance of prompt recognition and treatment of thyrotoxicosis to prevent severe cardiovascular sequelae.

## Introduction

Thyroid storm is an acute, life-threatening complication of hyperthyroidism characterized by an extreme hypermetabolic state with multisystem involvement [1]. It is an exacerbated form of thyrotoxicosis often precipitated by stressors such as infection, surgery, and trauma in patients with untreated or undiagnosed hyperthyroidism [1,2]. Fortunately, thyroid storm is uncommon, accounting for roughly 1-2% of hospital admissions for thyrotoxicosis. The disease continues to have a high death rate, which has been documented between 10% and 30% even with contemporary medical interventions [1]. Studies show that managing thyroid storm effectively reduces death rates to 5-10% [3], although this condition proves more fatal than typical thyrotoxicosis; however, thyroid storm still carries a significantly higher risk of death compared to uncomplicated thyrotoxicosis [4]. Prompt recognition and ICU-level care are therefore critical [2].

Graves’ disease, an autoimmune hyperthyroidism, occurs as a result of the immune system's production of thyroid-stimulating immunoglobulins that bind to and activate the thyroid-stimulating hormone (TSH) receptor on thyroid cells. This leads to thyroid gland hyperplasia and increased synthesis and release of T3 and T4 [5]. It is the most common cause of thyrotoxicosis in iodine-replete populations [5]. It has a peak incidence in middle age and a female predominance but can occur in males as well [6]. If left untreated, Graves’ hyperthyroidism can progress to thyroid storm under stress. Cardiovascular manifestations of thyrotoxicosis are prominent: patients often present with tachycardia, palpitations, widened pulse pressure, and arrhythmias such as atrial fibrillation, along with high cardiac output states [7]. In severe cases, heart failure (thyrotoxic cardiomyopathy) or angina can occur due to the profound metabolic demand [7]. Indeed, hyperthyroidism is associated with increased risk of cardiac events. One review stated that a small subset of patients (around 6%) initially present with myocarditis or overt heart failure before being diagnosed with thyrotoxicosis [8,9]. Here, we will present a case of thyroid storm secondary to previously undiagnosed Graves’ disease, which presented with striking cardiovascular complications mimicking acute coronary syndrome. The case highlights the importance of considering thyroid storm in patients with unexplained cardiac abnormalities and underscores the reversibility of cardiovascular dysfunction with appropriate therapy.

## Case presentation

**Figure 1:**
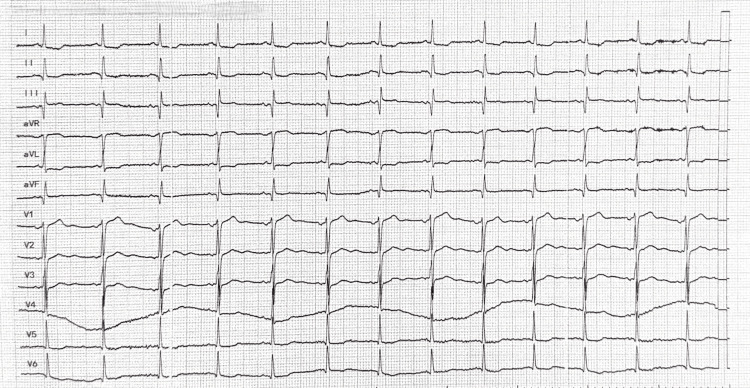
An electrocardiogram showing ST depressions in the leads I, AVL, V5, and V6.

A 50-year-old man with a known history of hypertension (on valsartan 80 mg daily) presented to the emergency department with an acute onset of severe chest pain, palpitations, and profuse diaphoresis of four hours duration. He also reported a one-week history of fever (reaching 38.7 degrees Celsius), diarrhea, and recurrent vomiting prior to admission. On arrival, he had signs of heart failure with fine crackles over lung bases and mild bilateral leg edema. Given his chest pain and tachyarrhythmia, cardiac enzymes were ordered: troponin I was elevated to 1.2 ng/mL (reference range <0.04 ng/mL), suggestive of myocardial injury. A focused ocular examination was performed and showed no signs of proptosis, pain with eye movement, chemosis, or lid lag suggestive of active thyroid orbitopathy. A bedside clinical activity score was estimated as 0, indicating the absence of inflammatory activity. An electrocardiogram revealed ST depressions in the lateral leads and non-specific ST-T changes (Figure 1). All initial laboratory findings are summarized in Table 1.

**Table 1:** Initial diagnostic laboratory profile. AST: Aspartate aminotransferase; ALT: alanine transaminase

Test	Value	Units	Reference Range
Hemoglobin	13.6	g/dL	13.5–17.5
White blood cell count	11.2	×10⁹/L	4.0–10.5
Platelets	250	×10⁹/L	150–400
Sodium	134	mmol/L	136–145
Potassium	3.9	mmol/L	3.5–5.1
Chloride	100	mmol/L	98–107
Bicarbonate	21	mmol/L	22–29
Blood urea nitrogen	18	mg/dL	7–20
Creatinine	0.9	mg/dL	0.6–1.3
Glucose	118	mg/dL	70–99
AST	42	U/L	0–40
ALT	50	U/L	0–45
Alkaline phosphatase	110	U/L	40–129
Total bilirubin	1	mg/dL	0.2–1.2
C-reactive protein	45	mg/L	<5
Troponin I	1.2	ng/mL	<0.04

**Figure 2:**
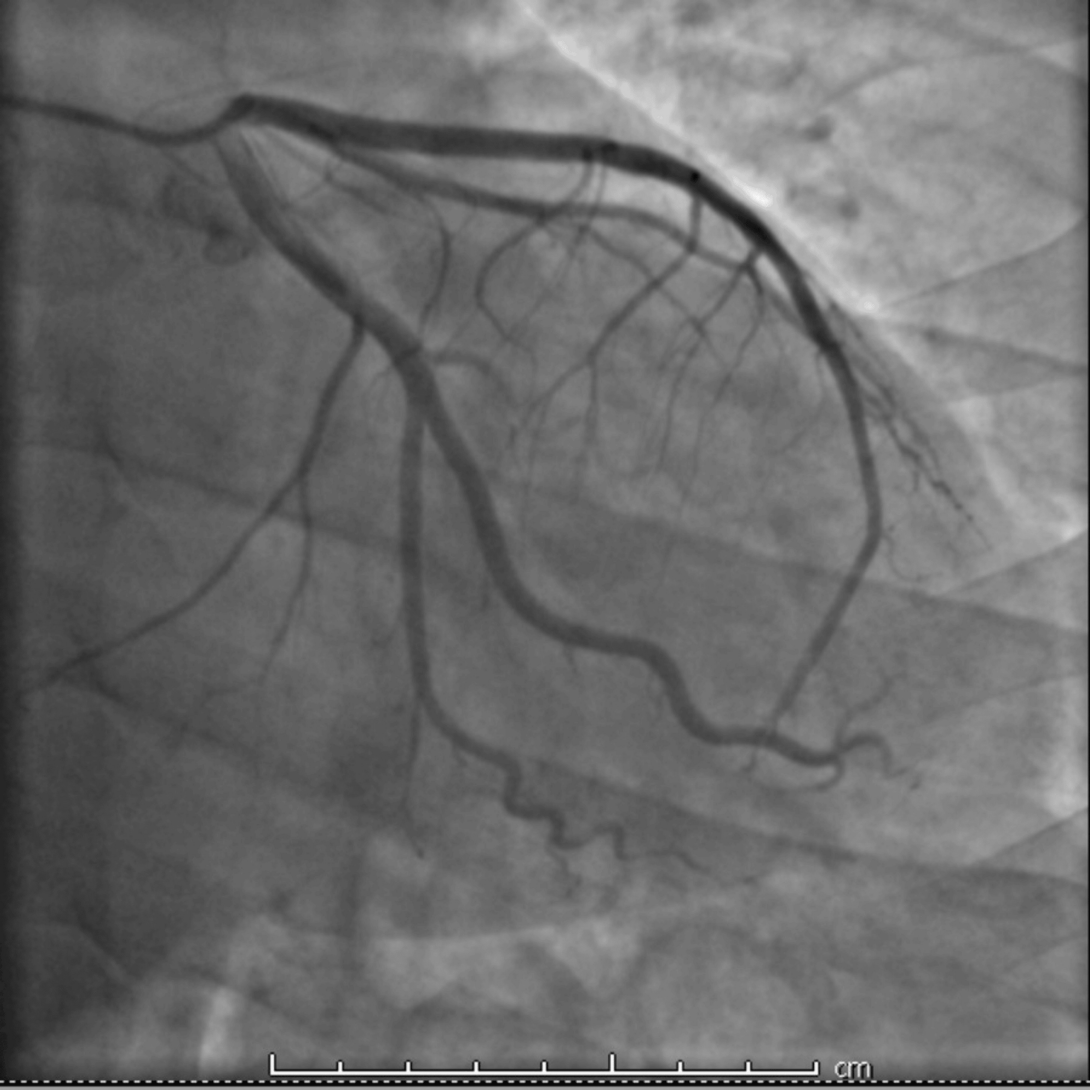
Coronary angiography of the left coronary system with no significant stenosis.

**Figure 3:**
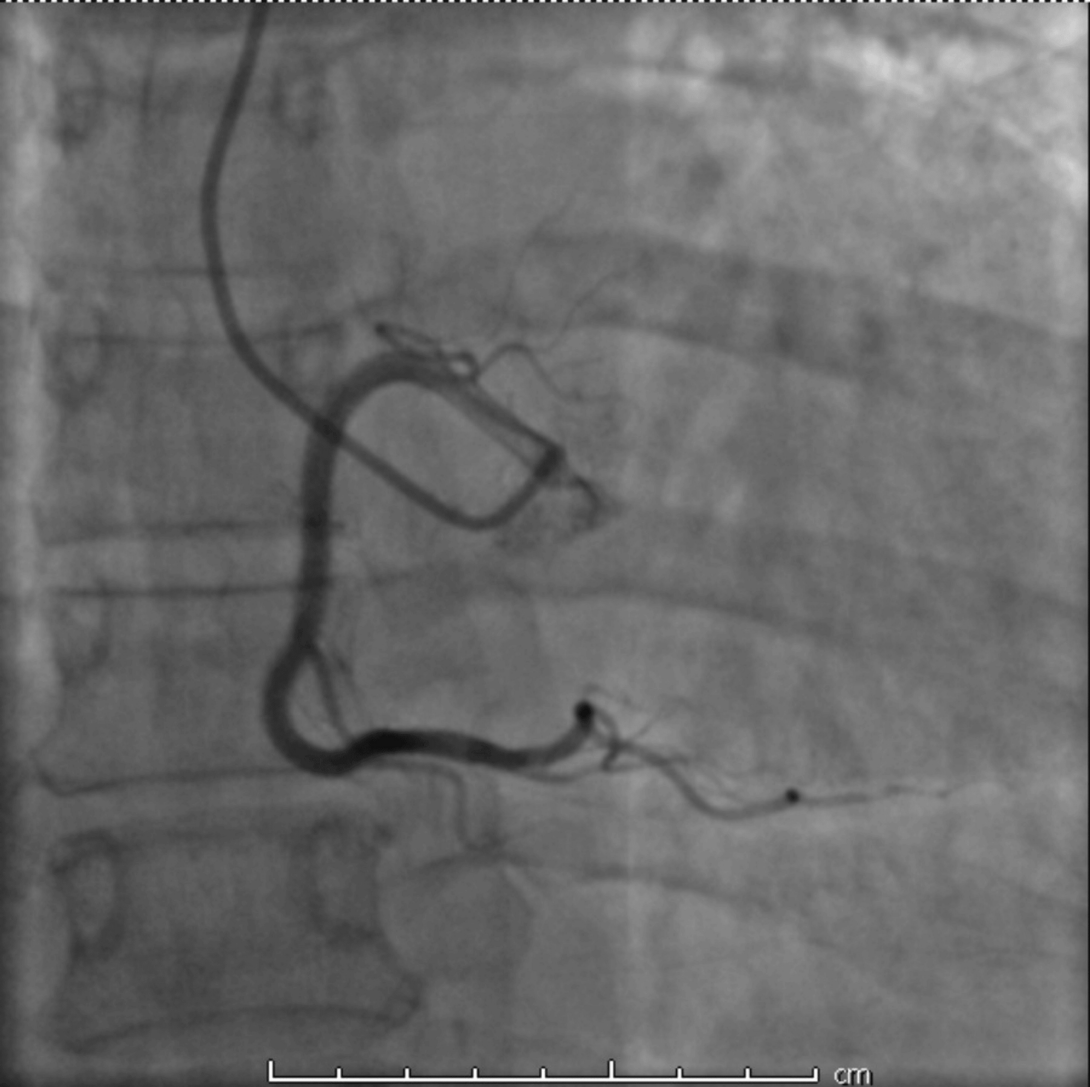
Coronary angiography of the right coronary system with no significant stenosis.

Transthoracic echocardiogram showed a hyperdynamic left ventricle with an ejection fraction of 65%, with grade I diastolic dysfunction, and mild left atrial enlargement measuring 42 mm in diameter. No regional wall motion abnormalities were noted. Based on the troponin rise and ECG findings, acute coronary syndrome was suspected. The patient was started on intravenous heparin, dual antiplatelet therapy, and a beta-blocker (metoprolol) while preparations were made for urgent coronary angiography, even with pending thyroid function tests. The patient underwent emergent coronary angiography, which revealed no significant occlusive coronary disease (Figures 2, 3).

Shortly after the procedure, the patient developed marked sinus tachycardia, with heart rate rising to 130-140 beats per minute (bpm), and his mental status worsened dramatically, becoming obtunded with a Glasgow Coma Scale of 8; thyroid function tests showed a suppressed TSH (<0.01 µIU/mL) and markedly elevated free T4 and T3 levels (free T4 >5 ng/dL, total T3 > 500 ng/dL; significantly above reference ranges). This mental deterioration was consistent with central nervous system (CNS) dysfunction due to severe thyrotoxicosis. Additional laboratory studies obtained at this time are presented in Table 2.

**Table 2:** Follow-up laboratory parameters during thyroid storm. NT-proBNP: N-terminal pro B-type natriuretic peptide

Test	Value	Units	Reference Range
TSH	<0.01	µIU/mL	0.4–4.0
Free T4	>5.0	ng/dL	0.8–1.8
Total T3	>500	ng/dL	80–180
Anti-TPO antibody	350	IU/mL	<35
TRAb	8	IU/L	<1
AST	60	U/L	0–40
ALT	72	U/L	0–45
Total bilirubin	1.3	mg/dL	0.2–1.2
Lactate	2.8	mmol/L	0.5–2.2
WBC	13.5	×10⁹/L	4.0–10.5
HCO3⁻	19	mmol/L	22–26
Serum cortisol	32	µg/dL	6–23
NT-proBNP	2500	pg/mL	<125 (<75 years)

The patient was thereafter transferred to the intensive care unit (ICU) and electively intubated for airway protection due to the decreased level of consciousness. Laboratory evaluation also revealed an elevated NT-proBNP level of 2,500 pg/mL (reference range <125 pg/mL for individuals under 75 years of age), consistent with volume overload and high-output cardiac strain secondary to thyrotoxicosis. At this point, the Burch-Wartofsky Point Scale (BWPS) was applied to evaluate the probability of thyroid storm. This scoring system assigns points based on thermoregulatory, cardiovascular, neurological, and gastrointestinal dysfunction, as well as the presence of precipitating factors. In this patient, the temperature of 38.7 degrees Celsius contributed 20 points, tachycardia exceeding 130 bpm contributed 20 points, CNS dysfunction with obtundation added 20 points, and the recent iodinated contrast exposure added another 10 points, giving a total BWPS of 70, well above the diagnostic threshold of 45, thus confirming the diagnosis of thyroid storm.

In the ICU, aggressive management for thyroid storm was initiated immediately. High-dose beta blockade was given with propranolol, titrated to 80 mg orally via a nasogastric tube (NGT) every six hours to control tachycardia and hyperadrenergic symptoms. Thionamide therapy was started with propylthiouracil (PTU) in a high-dosage regimen, a loading dose of 600 mg via NGT, followed by 250 mg every four hours around the clock. Approximately one hour after the first PTU dose, iodine solution was administered in the form of Lugol’s iodine (five drops, roughly 8 mg per drop, given orally every six hours) in order to inhibit further thyroid hormone release. (It is critical to give iodine at least an hour after thionamide initiation to prevent new hormone synthesis from the iodine load.) Glucocorticoid therapy was also added, with intravenous hydrocortisone 100 mg every eight hours used to reduce peripheral T4 to T3 conversion and treat potential relative adrenal insufficiency. Supportive care measures included active cooling with an ice blanket for fever, intravenous fluids with dextrose, and sedation along with analgesia while intubated. Empiric broad-spectrum antibiotic therapy with intravenous piperacillin-tazobactam (4.5 g every eight hours) was started because infection was considered a possible precipitating factor, given the patient’s fever and markedly elevated inflammatory markers. These antibiotics were discontinued once infection was excluded. Further workup confirmed Graves’ disease as the etiology of his thyrotoxicosis. Anti-thyroid peroxidase (anti-TPO) antibody was significantly elevated at 350 IU/mL (normal <35), and thyrotropin receptor antibody (TRAb) titer was also reported positive at 8 IU/L (normal <1). A radioactive iodine uptake scan performed after stabilization showed diffusely increased uptake throughout the thyroid gland, confirming the diagnosis of Graves’ disease. These findings corroborated that his thyroid storm was precipitated by undiagnosed Graves’ hyperthyroidism.

The patient remained in sinus tachycardia during his ICU stay; however, no atrial fibrillation was noted on telemetry. By day 3, the patient was extubated, and his thyroid function tests showed improvement (free T4 down to 2.5 ng/dL) and his clinical storm symptoms abated. The patient was transitioned from PTU to methimazole 30 mg daily after the thyroid storm resolved, and the beta-blocker was changed to oral propranolol. He was discharged home after one week in stable condition, on a regimen of methimazole, propranolol, and corticosteroid taper. As no active thyroid orbitopathy was present on examination, radioiodine ablation was considered safe and was chosen as the definitive treatment.

One month later, he underwent radioiodine ablation. On follow-up three months post-RAI, he had become clinically and biochemically euthyroid with complete resolution of his symptoms. Notably, his cardiac findings had fully normalized, a repeat echocardiogram showed normal chamber sizes and an ejection fraction of 65% with no segmental wall motion abnormalities, and he reported no further chest pain or arrhythmias. This outcome suggested that the cardiovascular dysfunction was reversible and secondary to the hyperthyroid state.

## Discussion

Thyroid storm represents the most severe manifestation of hyperthyroidism. It remains a life-threatening endocrine emergency [1,2]. This condition develops when thyroid hormone levels exceed normal ranges, which leads to multi-organ failure [2]. The cardiovascular system is particularly vulnerable to thyrotoxic stress; thus, the heart experiences increased contractility associated with tachycardia and an increase in oxygen consumption due to thyroid hormones, which simultaneously lower systemic vascular resistance to produce a high-output hyperdynamic state [7]. As a result, common cardiovascular findings include sinus tachycardia, atrial arrhythmias, widened pulse pressure, and, in some cases, high-output heart failure [7]. In our patient, persistent tachycardia and high-output circulatory effects led to acute chest pain with ECG changes and elevated troponin, initially suggesting an acute coronary syndrome. Importantly, NT-proBNP was markedly elevated at 2,500 pg/mL (reference <125 pg/mL), supporting volume overload and high-output cardiac strain rather than primary systolic dysfunction. The echocardiogram findings of a hyperdynamic left ventricle with preserved ejection fraction and only mild diastolic dysfunction further reinforce that the cardiac injury was functional and reversible.

Importantly, thyroid storm can lead to significant troponin elevation and ischemic-appearing ECG changes despite the absence of coronary artery obstruction. This reflects a type II myocardial infarction mechanism, in which the myocardial oxygen demand exceeds supply under the intense hypermetabolic and adrenergic stress of thyrotoxicosis [9]. Excess thyroid hormone increases heart rate, contractility, and oxygen consumption and may trigger transient coronary vasospasm, collectively leading to reversible myocardial ischemia even with normal coronary anatomy [9,10]. Our patient’s marked troponin elevation and lateral ST depressions, coupled with a normal coronary angiogram, strongly support this mechanism. Following prompt thyroid-directed therapy, both his chest pain and cardiac biomarkers normalized, confirming demand-related ischemia rather than acute plaque rupture. Differentiating type II MI (secondary to hyperthyroid crisis) from a type I MI (primary ACS) is crucial, as treatment priorities differ [9]. The main treatment for thyroid storm requires immediate control of thyrotoxicosis, whereas indiscriminate use of anticoagulation or invasive coronary intervention may be unnecessary if clinical and diagnostic data support a secondary cause [11]. Some reported cases showed patients with thyrotoxic crisis with critical coronary stenoses requiring stenting; thus, clinicians should evaluate for acute coronary syndrome in thyroid storm (especially in older patients or those with risk factors) and manage accordingly until a primary cardiac event is ruled out [11]. Another key factor in this case is the precipitating role of iodinated contrast exposure during coronary angiography. The Jod-Basedow phenomenon occurs when iodine intake triggers excessive thyroid hormone production and release in people who have latent Graves’ disease [12]. Contrast-induced thyrotoxicosis has been reported to precipitate thyroid storm, particularly in patients with unrecognized autoimmune hyperthyroidism [12]. Our patient’s deterioration shortly after angiography strongly suggests this mechanism contributed to the crisis.

Another significant cardiac complication of severe thyrotoxicosis is thyrotoxic cardiomyopathy, a form of dilated cardiomyopathy resulting from prolonged tachycardia and myocardial strain [6]. Our patient did not develop overt heart failure, despite having early signs of cardiomyopathic changes (mild diastolic dysfunction and atrial enlargement on echo). Thyrotoxic cardiomyopathy is uncommon, with an estimated 1% in patients with hyperthyroidism. Encouragingly, when recognized and treated, thyrotoxic cardiomyopathy is often reversible [6]. Restoring a euthyroid state allows the myocardium to recover in many cases. For example, Malani et al. reported an elderly patient diagnosed with Graves’ disease who developed severe dilated cardiomyopathy (EF 25%), who completely recovered with normal cardiac function after appropriate antithyroid therapy [6]. Our patient’s cardiac function likewise normalized after treatment of his hyperthyroidism. This reversibility highlights the critical importance of early diagnosis and management of thyrotoxicosis in patients presenting with new-onset heart failure. In addition, hyperthyroid-related tachyarrhythmias (such as atrial fibrillation) often resolve once the euthyroid state is achieved, obviating long-term arrhythmia therapy in many cases [6].

The management of cardiovascular complications in thyroid storm needs both supportive care and controlling thyroid hormone effects [13]. Beta-blockers function as the main treatment because they help manage the heart rate, decrease contractility, and stop peripheral conversion of T4 to T3 [13]. Non-selective agents such as propranolol are preferred for their combined cardiac and metabolic benefits [13].

Lim et al. have documented precipitous circulatory collapse in thyroid storm after beta-blocker initiation in patients with underlying cardiac depression; therefore, caution is advised in patients with cardiogenic shock or heart failure, since excessive beta-blockade may worsen cardiac output and trigger hemodynamic collapse [2]. Titration and close monitoring are essential, and if needed, short-acting agents or partial agonists can be employed [2]. In refractory cases, extracorporeal support has been used as a bridge while controlling thyrotoxicosis [2].

The most effective cardiac treatment for our patient emerged from treating the thyrotoxicosis condition directly. The immediate use of antithyroid medications together with iodine in thyroid storm treatment blocks hormone production and release, which helps to decrease the sudden increase in heart metabolic needs. Glucocorticoids, including hydrocortisone, function to decrease T4-T3 conversion while simultaneously treating relative adrenal insufficiency [13]. Supportive care, cooling methods, fluid administration, and trigger management (such as antibiotic treatment for infections) play a vital role in treatment [13]. Our patient received this combination of treatments, which led to progressive improvement. Notably, no anti-arrhythmic drugs were needed in our case since no sustained atrial fibrillation occurred.

Once a thyroid storm patient is stabilized, attention must turn to definitive therapy for the underlying hyperthyroidism. Recurrence of thyroid storm is a major concern if hyperthyroidism persists. So radioactive iodine ablation or thyroidectomy is recommended in case of recurrence [14]. In our patient’s case, we proceeded with radioiodine ablation one month following the storm. This achieved definitive resolution of his Graves’ disease. Following definitive therapy, his risk of another thyroid storm is essentially decreased. Reports indicate that cardiomyopathy or heart rhythm disturbances caused by thyrotoxicosis do not typically recur after the hyperthyroid state is permanently abolished [14]. Indeed, our patient’s cardiac exam and echocardiogram went back to normal on follow-up.

It is important to note that thyroid storm can precipitate less common cardiac phenomena as well. For instance, there are documented cases of Takotsubo (stress) cardiomyopathy triggered by hyperthyroid crisis [15]. This presents as acute heart failure with apical ballooning of the left ventricle, mimicking an acute MI, but is reversible [16]. Clinicians should be aware of this possibility, as hyperthyroidism can be a reversible cause of Takotsubo syndrome.

## Conclusions

Thyroid storm should be recognized as a rare but critical cause of acute decompensation, especially when patients present with cardiovascular collapse or acute coronary syndrome features out of proportion to their risk factors. This case demonstrates that thyroid storm can mimic myocardial infarction and heart failure, yet prompt appropriate therapy can lead to complete reversal of cardiac abnormalities. Physicians must rapidly initiate multi-faceted treatment like beta-blockers, antithyroid drugs, iodine, steroids, and supportive care in order to stabilize the patient.

Moreover, iodinated contrast exposure can precipitate or even worsen thyrotoxicosis in undiagnosed Graves’ disease, emphasizing the need for vigilance in hyperthyroid-prone patients undergoing contrast studies. Definitive treatment of the underlying hyperthyroidism (with radioiodine or surgery) is essential to prevent recurrence of thyroid storm. With timely management, even severe thyrotoxic cardiac complications are largely reversible, and patients can expect good cardiac outcomes once a euthyroid state is restored.
